# Melatonin Attenuates Chromium (VI)-Induced Spermatogonial Stem Cell/Progenitor Mitophagy by Restoration of METTL3-Mediated RNA N^6^-Methyladenosine Modification

**DOI:** 10.3389/fcell.2021.684398

**Published:** 2021-06-04

**Authors:** Yinghua Lv, Tianjiao Li, Manman Yang, Lihong Su, Zhendong Zhu, Sihang Zhao, Wenxian Zeng, Yi Zheng

**Affiliations:** ^1^Shaanxi Key Laboratory of Natural Products & Chemical Biology, College of Chemistry & Pharmacy, Northwest A&F University, Yangling, China; ^2^Key Laboratory for Animal Genetics, Breeding and Reproduction of Shaanxi Province, College of Animal Science and Technology, Northwest A&F University, Yangling, China; ^3^College of Animal Science and Technology, Qingdao Agricultural University, Qingdao, China

**Keywords:** chromium (VI), spermatogonial stem cells, mitophagy, melatonin, RNA m^6^A modification

## Abstract

Spermatogonial stem cells (SSCs) are the basis of spermatogenesis, and any damage to SSCs may result in spermatogenic disorder and male infertility. Chromium (Cr) (VI) is a proven toxin, mutagen, and carcinogen, perpetually detrimental to environmental organisms due to its intricate and enduring detoxification process *in vivo*. Despite this, the deleterious effects of Cr (VI) on SSCs and the underlying mechanisms remain poorly understood. In this study, we identified that Cr (VI) impaired male reproductive system in mouse testes and induced mitochondrial dynamic imbalance and mitophagy in SSCs/progenitors. Cr (VI) also downregulated the RNA N^6^-methyladenosine (m^6^A) modification levels in mitochondrial dynamic balance and mitophagy genes in SSCs/progenitors. Inspiringly, the toxic effects of Cr (VI) could be relieved by melatonin pretreatment. Melatonin alleviated Cr (VI)-induced damage to male reproductive system and autophagy in mouse testes. Melatonin also attenuated Cr (VI)-induced cell viability loss and reactive oxygen species (ROS) generation, as well as mitochondrial dynamic disorders and mitophagy in SSCs/progenitors. The protective roles of melatonin against Cr (VI)-induced mitophagy were exerted by restoration of METTL3-mediated RNA m^6^A modification and activation of mitochondrial fusion proteins MFN2 and OPA1, as well as inhibition of the mitophagy BNIP3/NIX receptor pathway. Thus, our study provides novel insights into the molecular mechanisms for RNA m^6^A modification underlying the gene regulatory network responsible for mitochondrial dynamic balance, and also lays new experimental groundwork for treatment of Cr (VI)-induced damage to male fertility.

## Introduction

Chromium (Cr) is a natural metal element and most frequently detected in soil, ground water, air, rocks, and living organisms. Chromium and chromium-containing compounds are prevalent, mainly due to their extensive industrial applications, such as electroplating, leather tanning, stainless steel production, water cooling, printing inks, textile dyeing, pigment manufacturing of wood, drilling muds, fireworks, wood preservation, and anti-corrosion and conversion coatings (Ukhurebor et al., 2021). Trivalent chromium [Cr (III)] and hexavalent chromium [Cr (VI)] are the two stable valence states typically found in workplace and environment (Kotas and Stasicka, 2000; Costa, 2003). In natural systems, Cr (III) is generally considered as low toxicity and mobility in humans and animals (Chen et al., 2019). By contrast, Cr (VI) is a strong oxidizing agent and one of the most toxic environmental potential carcinogens. Cr (VI) is toxic to both plants and animals, perpetually detrimental to environmental organisms due to its intricate and enduring detoxification process *in vivo* (Zhitkovich, 2011). The well-known cellular toxicity of Cr (VI) can be attributed to reactive oxygen species (ROS) generation that triggers oxidative stress, DNA damage, genomic instability (Zhitkovich, 2005), or epigenetic modulation (DesMarais and Costa, 2019). While the potential toxicity of Cr (VI) has appealed to the public, its deleterious effects on male fertility remain to be explored. For instance, the detrimental influences of Cr (VI) on spermatogonial stem cells (SSCs) warrant systematic investigation. Since SSCs are the cornerstone of spermatogenesis and able to differentiate into sperm thereby transmitting paternal genetic information to the next generation (Kubota and Brinster, 2018; Sharma et al., 2019), studies in this respect would be of great value to male fertility and reproductive health.

Epigenetic events, including DNA methylation and histone modifications, regulate SSC homeostasis (Makela and Hobbs, 2019). Our previous study showed that high dosages of Cr (VI) induced a global increase of H3K9me3 and activated apoptotic signaling pathways in mouse SSCs (Lv et al., 2018). Whether this phenotype also involves changes in other epigenetic markers, e.g., N^6^-methyladenosine (m^6^A) modification of RNA methylation, remains elusive. RNA m^6^A modification is the most abundant and widely distributed RNA modification in eukaryotes, accounting for more than 90% in all RNA modifications (Meyer et al., 2012). It is involved in RNA splicing, translation, stabilization, and degradation, thus playing regulatory roles in many biological processes, including SSC maintenance and spermatogenesis (Hsu et al., 2017; Lin et al., 2017; Xu et al., 2017). Previous studies have shown that activation of stress-response pathways causes global reshaping of the cellular mRNA methylome. More precisely, in the heat shock state, m^6^A residues within the 5' UTR promote cap-independent translation of heat shock factor (HSF) mRNAs, activating the cell heat shock response pathway (Meyer et al., 2015). Additionally, under UV irradiation, RNA m^6^A modification could recruit DNA repair enzyme pol κ to DNA damage sites to facilitate DNA repair and cell survival (Xiang et al., 2017). These studies suggest that RNA m^6^A modification is involved in regulation of gene expression and in protection of cells under stress conditions. It has also been reported that m^6^A is essential for the well-being of male reproductive system. For instance, the increased m^6^A modification of RNA methylation is related to the inhibition of demethylase FTO thereby contributing to MEHP-induced Leydig cell injury (Zhao et al., 2021). However, it remains unknown whether RNA m^6^A modification is involved in Cr (VI)-induced toxicity in SSCs.

Melatonin is an endogenous indoleamine hormone. As the strongest endogenous free radical scavenger of ROS and inducer of antioxidant systems *in vivo* (Bonnefont-Rousselot et al., 2011), melatonin is involved in various physiological processes, such as apoptosis and autophagy in cancer cells, neurodegeneration and progressions of liver diseases as well as other pathologies (Fernandez et al., 2015). Recent studies by peers and us have shown that melatonin could protect testes against heat-induced damage (Zhang et al., 2020a) and spermatogonia against the stress of chemotherapy and oxidation *via* elimination of ROS (Zhang et al., 2019), and that melatonin also has protective roles against Cr (VI)-induced apoptosis and the global levels of H3K9me3 and H3K27me3 in mouse SSCs (Lv et al., 2018). Nevertheless, whether melatonin has effects on Cr (VI)-induced RNA m^6^A modification in SSCs and, if any, the underlying mechanisms remain to be probed.

In this study, we first demonstrated that Cr (VI) impaired male reproductive system in mouse testes, along with a decrease in cell viability but an increase in ROS generation in SSCs/progenitors. Then, we identified that RNA m^6^A modification was involved in Cr (VI)-induced mitochondrial dynamic disorders and mitophagy. Inspiringly, melatonin alleviated Cr (VI)-induced damage to male reproductive system and autophagy in mouse testes. Melatonin also attenuated Cr (VI)-induced cell viability loss and ROS generation, as well as mitochondrial dynamic disorders and mitophagy in SSCs/progenitors. The protective roles of melatonin against Cr (VI)-induced mitophagy were exerted by restoration of METTL3-mediated RNA m^6^A modification and activation of mitochondrial fusion proteins MFN2 and OPA1, as well as inhibition of the mitophagy BNIP3/NIX receptor pathway. Thus, our study provides novel insights into the molecular mechanisms for RNA m^6^A modification underlying the gene regulatory network responsible for mitochondrial dynamic balance, and also lays new experimental groundwork for treatment of Cr (VI)-induced damage to male fertility.

## Materials and Methods

### Chemicals and Antibodies

Na_2_CrO_4_ (98% of purity; 307,831) and melatonin (98% of purity; M5250) were purchased from Sigma-Aldrich (St. Louis, MO, United States). The detailed antibody information is shown in Table 1.

**Table 1:** The detailed antibody information.

Antibody	Species source	Supplier	Identifier	Dilution	
				WB	IHC
LIN28	Rabbit	Abcam	ab46020		1:200
CDH1	Rabbit	Proteintech	20874-1-AP		1:200
m^6^A	Rabbit	Synaptic systems	202003	1:1,000	
Beclin1	Rabbit	Cell signaling technology	3738s	1:1,000	
p-Beclin1	Rabbit	Cell signaling technology	13825s	1:1,000	
LC3B	Rabbit	Sigma-Aldrich	L-7543	1:1,000	
β-actin	Mouse	CWBIO	CW0096	1:2,000	
Tom20	Rabbit	Proteintech	11802-1-AP	1:1,000	
MT1	Mouse	Abnova	H00004543-A01	1:1,000	
NOX4	Rabbit	NOVUS	NB110-58849	1:1,000	
METTL3	Rabbit	Cell signaling technology	15073-1-AP	1:1,000	

WB, western blot; IHC, immunohistochemistry.

### Cells and Treatment

Immortalized mouse SSCs/progenitors (the C18-4 cell line) were established from type A spermatogonia in testes from 6-day-old mice (Hofmann et al., 2005). The cells were cultured with the medium comprising DMEM (high glucose, Hyclone), 10% FBS (BI) and 1% penicillin-streptomycin (Invitrogen, 15140122). Cells were maintained at 37°C in an atmosphere of 5% CO_2_ in air. Cells were pretreated with vehicle or 50 μM melatonin for 2 h, followed by treatment with 10 μM Cr (VI) for 1 or 4 h, unless otherwise stated.

### Animals and Treatment

C57BL/6J mice were purchased from the laboratory animal center of the Fourth Military Medical University, Xi’an, China. Adult male mice aged 8–10 weeks, with the body weight (*b.w.*) 22–25 g, were fed with the basal diet and kept at 20 ± 2°C and 50 ± 5% humidity under a 12-h light-dark cycle. Forty mice were divided into four groups at random, with 10 mice in each group: the control group (intraperitoneal injection with an equal volume of physiological saline daily), the Cr (VI) group [intraperitoneal injection with 16.2 mg/kg *b.w.*/day Cr (VI)], the melatonin + Cr (VI) group [intraperitoneal injection with 25 mg/kg *b.w.*/day melatonin at 4 h before intraperitoneal injection with 16.2 mg/kg *b.w.*/day Cr (VI)] and the melatonin group (intraperitoneal injection with 25 mg/kg *b.w.*/day melatonin). Melatonin and Cr (VI) treatment lasted for 14 consecutive days, followed by 14 days of recovery before sacrifice. All animal procedures were in accordance with and approved by the Institutional Animal Care and Use Committee of Northwest A&F University (DK-314020038).

### Hematoxylin and Eosin Staining

Hematoxylin and eosin (H&E) staining was performed as previously reported (Zheng et al., 2019). Briefly, after deparaffinization and rehydration, testis sections were stained with hematoxylin and eosin, and then sealed with neutral gum and examined under a light microscope. To quantify the ratios of only 1–3 layers of germ cell, empty and abnormal tubules, 300 seminiferous tubules from five mice were analyzed in each group.

### Immunohistochemistry

Mouse testes were collected, fixed in diluted Bouin’s solution and embedded in paraffin. Testis sections were sliced at a thickness of 6 μm. After deparaffinization and rehydration, testis sections were permeabilized with 0.5% Triton X-100 (Solarbio) for 10min and subjected to heat-induced antigen retrieval in 10 mM sodium citrate buffer (pH = 6.0), followed by blocking with 10% donkey serum for 2 h at room temperature. Testis sections were then incubated with primary antibodies (Table 1) at 4°C overnight. Next day, testis sections were washed with PBS and incubated with the corresponding secondary antibody anti-rabbit-Alexa Fluor 488 (1:300; 711-545-152, Jackson Immunoresearch) or anti-rabbit-Alexa Fluor 594 (1:300; 711-585-152, Jackson Immunoresearch) for 1 h at 37°C. After washing, testis sections were stained with DAPI (1:1,000; BioWorld) and examined under a fluorescence microscope (Nikon Eclipse 80i, Tokyo, Japan).

### Assays of Sperm Number, Progressive Motility, and Morphological Abnormality

Sperm progressive motility was determined by computer-assisted sperm analysis (CASA; HVIEW, China), as previously reported (Li et al., 2020a). In brief, the sample (0.5 ml) was pre-incubated at 37°C for 5 min, and the sperm progressive motility was defined as the percentage of spermatozoa with straight line velocity (VSL) > 25 μm/s and straightness of path (STR) ≥ 75%. The standard parameter was set at 30 frames/s. A minimum of 300 spermatozoa were observed from at least five randomly selected fields with 20 μm CELL-VU® DRM-600 sperm count slides (Millennium Sciences, United States) and a microscopic stage warmer (KITAZATO, Japan). Later, the sample was fixed with ethanol, and the sperm number was evaluated using CASA and 20 μm CELL-VU® DRM-600 sperm count slides. Then, the mouse semen sample was fixed in 4% paraformaldehyde for 24 h and subsequently spread on slides. H&E staining was conducted for sperm morphology examination. Over 1,000 spermatozoa were examined for morphological abnormality under a light microscope, and assessment of sperm morphological abnormality was referred to a previous report (Gotoh et al., 2012).

### Depletion of Mettl3 by Lentivirus-Mediated shRNA Targeting

To stably deplete *Mettl3* in mouse SSCs/progenitors, lentiviruses harboring shRNA targeting mouse *Mettl3* were packaged, produced, and delivered to cells, following a previous article (Zheng et al., 2020). In brief, lentiviruses were produced by co-transfecting the cloned shRNA expressing vectors (backbone: pGreenPuro; System Biosciences) and the 2nd generation packaging vectors psPAX2 and pMD2.G into HEK293T cells, and were concentrated using ultracentrifugation. Mouse SSCs/progenitors exposed to 10 μg/ml polybrene (Sigma-Aldrich) and the concentrated virus supernatant at a multiplicity of infection (MOI) of 20 were centrifuged at 3,000 *g* for 1 h at 32°C, followed by 16 h of incubation at 37°C. About 5 days after lentiviral transduction, cells were harvested for validation or downstream experiments. For construction of the shRNA expressing vectors, a sequence specific to the mouse *Mettl3* cDNA (5'-GCTACCGTATGGGACATTA-3') or a scramble sequence (5'-GACACCTACGCAAAACCCT-3') was used.

### CCK-8 Assay

Cell viability was determined using a Cell Counting Kit-8 (CCK-8) assay kit (Beyotime, Beijing, China). Mouse SSCs/progenitors were prepared and dispersed in 96-well cell culture plates at a density of 1.0 × 10^4^ cells/well. After overnight incubation, cells were either exposed to Cr (VI) at the dose of 0, 2.5, 5, 10, 25, or 50 μM for 12 h, or to 10 μM Cr (VI) for 0, 1, 4, 8, 12, 16, 20, or 24 h, unless otherwise stated. Then, about 10 μl of the CCK-8 solution diluted in DMEM was added to each well and incubated for 1.5 h at 37°C. The optical density of each well was measured at 490 nm with a microplate reader. Three independent experiments were performed, and in each independent experiment, at least three parallel measurements were performed.

### MTT Assay

Mouse SSCs/progenitors were prepared and dispersed in 96-well cell culture plates at a density of 1.0 × 10^4^ cells/well. After overnight incubation, cells were treated with Cr (VI) at the dose of 0, 2.5, 5, 10, 25 or 50 μM for 24 h. Then, the cells were washed with PBS, and the fresh medium containing MTT [3-(4,5)-dimethylthiahiazo (-z-y1)-3,5-di-phenytetrazoliumromide] was added to each well, followed by 4 h of incubation. Later, the medium containing MTT was removed, and dimethyl sulfoxide (100 μl) was added to each well. The plate was then gently shaken for 10 min to dissolve formazan crystals. Finally, the absorbance at 490 nm in each well was recorded with a microplate reader. Three independent experiments were performed, and in each independent experiment, at least three parallel measurements were performed.

### EdU Assay

Mouse SSCs were prepared and dispersed in 96-well cell culture plates at a density of 1.0 × 10^4^ cells/well. After overnight incubation, cells were exposed to Cr (VI) at the dose of 0, 2.5, 5, 10, 25, or 50 μM for 24 h. Then, the cells were washed with PBS, and subjected to an EdU assay, as previously reported (Zheng et al., 2017). Three independent experiments were performed.

### MDC Incorporation Assay

A fluorescent compound, namely monodansylcadaverine (MDC; Solarbio), has been proposed as a tracer for autophagic vacuoles. Thus, autophagic vacuoles can be detected by MDC staining. After the melatonin and/or Cr (VI) treatment, cells were incubated with 50 μM MDC in a serum-free medium for 30 min at 37°C. Then the cells were washed with PBS for three times and fluorescence micrographs were captured using an inverted fluorescence microscope (Olympus IX71, Tokyo, Japan).

### m^6^A Dot-Blot Assay

Total RNAs were extracted from cells with Trizol reagent (Takara) and mRNAs were purified using PolyATtract® mRNA Isolation Systems (Promega, Z5310) following the manufacturer’s instructions. Briefly, mRNA samples were loaded onto a Hybond-N^+^ membrane (GE HealthCare, RPN303B) and crosslinked to the membrane with UV radiation. Then, the membrane was blocked with 5% non-fat milk (Bio-Rad) for 2 h, followed by incubation with a rabbit anti-m^6^A polyclone antibody (Synaptic Systems, 202003) at 4°C overnight. Next day, the membrane was incubated with an HRP-conjugated goat anti-rabbit IgG (CWbio, CW0156) antibody for 2 h at room temperature. The immunocomplex was visualized and captured by a Bio-Rad Chemidoc XRS with a Western Bright ECL Kit (Bio-Rad, Berkeley, CA, United States). Finally, the membrane was stained with 0.02% methylene blue to eliminate the difference in mRNA amount.

### Mito and Lyso Tracker Staining

Cells were pretreated with vehicle or 50 μM melatonin for 2 h, followed by treatment with 10 μM Cr (VI) for 1 or 4 h. Then, the cells were refreshed and incubated with 200 nM Mito-Tracker Green (Beyotime, Beijing, China) and 75 nM Lyso-Tracker Red (Beyotime, Beijing, China) for 45 min. Subsequently, the cells were washed twice and visualized under an inverted microscope (Olympus IX71, Tokyo, Japan). To quantify the ratio of cells double positive for both Mito and Lyso staining, 100 cells were analyzed in each group and three independent experiments were performed.

### Mitochondrial Membrane Potential Assay

Mitochondrial membrane potential (MMP) was determined by using the lipophilic cationic dye JC-1 (Beyotime, Beijing, China). Cells were seeded into 96-well plates with a density of 1 × 10^4^ cells/well. After overnight plating, cells were pretreated with vehicle or 50 μM melatonin for 2 h, followed by treatment with 10 μM Cr (VI) for 1 or 4 h. After washing, the cells were incubated with JC-1 for 20 min at 37°C. Then a microplate reader was employed to detect the fluorescence intensity. The ratio of red/green fluorescence density indicated mitochondrial polarization.

### Analysis of the ROS Level

The intracellular ROS accumulation was detected by the ROS Assay Kit (Beyotime, Beijing, China). Cells were seeded to 6-well plates and treated with Cr (VI). Then the cells were incubated with 10 μM DCFH-DA for 20 min at 37°C, dissociated by trypsin, and collected after centrifugation. The same number of cells were seeded into 96-well plates, and detected by a microplate reader.

### m^6^A-IP-qPCR Analysis

Quantitative real-time PCR (qPCR) analyses were performed to detect the relative abundance of the selected mRNA in the m^6^A antibody IP sample and in the input sample. Briefly, total RNAs were extracted from cells using the RNAiso plus reagent (Takara, Dalian, China). mRNAs were purified from total RNAs using the PolyATtract mRNA Isolation Systems (Promega, Z5310) and then fragmented using RNA Fragmentation reagent (Invitrogen, AM8740) for 1 min at 94°C. Protein A beads were washed and diluted into 500 μl IP buffer (150 mM NaCl, 0.1% NP-40, 10 mM Tris, pH = 7.4, 100 U RNase inhibitor) and incubated with a m^6^A antibody (Synaptic Systems, 202003) for 1 h at 4°C. About 10% of the fragmented RNAs were left aside as input RNAs, whereas the remaining RNAs were added to the mixture and incubated for 4 h at 4°C with rotation. The mRNAs harboring m^6^A were eluted using 100 μl elution buffer (IP buffer, 6.7 mM m^6^A) for 1 h at 4°C and precipitated with 5 mg glycogen (Life Technologies, AM9510) and one-tenth volume of 3 M sodium acetate (Solarbio) in 2.5 volumes of 100% ethanol at −80°C overnight. The same numbers of the concentrated IP RNAs or input RNAs from each sample were used for cDNA synthesis. The m^6^A enrichment was finally determined by qPCR analysis.

### qPCR Analysis

Total RNAs were extracted from cells with Trizol reagent (Takara, Dalian, China), according to the manufacturer’s protocol. For each sample, 1 μg total RNAs were subjected to reverse transcription into cDNAs using the PrimeScript™ RT reagent Kit with gDNA Eraser (Takara, Dalian, China). qPCR analyses were conducted with an IQ5 (Bio-Rad, Berkeley, CA, United States). Reactions were run in triplicates, and three independent experiments were performed. The geometric mean of the housekeeping gene *β-actin* was used as an internal reference and the data were analyzed using the 2^-△△Ct^ method. Primer sequences for qPCR analyses were shown in Table 2.

**Table 2:** Primer sequences for qPCR analyses.

Gene	Forward primer (5'→3')	Reverse primer (5'→3')	Product size, bp
*Mfn1* *Mfn2* *Opa1* *Drp1* *Bnip3*	AGAGCCCATCTTTCAGGTCC AGCAGATTACGGAGGAAGTGGA GCTTCAAGGTCGTCTCAAGGATA CAGTGTCCCAAAGGCAGTAATG TTCAGCAATGGCAATGGGA	TTAGTTTCCAGCCCACTGTTTTC GAGCAGCGGTCAGACAGGTT TCGTTCTTGGTTTCGTTGTGA CGCTGCTTCTTTTCTTCGTTG TTGTGGTGTCTGGGAGCGA	197 190 129 151 153
*Nix*	GCAATGAGAATGGAAATGGGA	TTCTTGTGGTGAAGGGCTGTC	164
*Mettl3* *Wtap* *Fto*	GAGGTTCGTTCCACCAGTCATAA AGTTATGGCACGGGATGAGTTA CTGAGGATGAAAGTGAGGACGAG	TAGGTTTAGAGATGATGCCGTCC TCCTGCTGTTGCTGCTTTAGTT TGGAACTAAACCGAGGCTGTG	217 147 204
*Ythdf2* *β-actin* *Mfn2*-IP *Opa1*-IP *Bnip3*-IP *Nix*-IP	CCTCTTGGAGCAGAGACCAAA TCTTTTCCAGCCTTCCTTCTTG ATGTGTCTGTGTCTGCTCCTCA AGGTCATCAGTCTGAGCCAGGT CTGCCCCTGCTACCTCTCG CTTCCTCGTCTTCCATCCACA	TTATTCGGCCTTGCCTGTGG GTTGGCATAGAGGTCTTTACGGA TGTGCTCAGGCTGGAGAAAGTA CTGTGGTGTTAAATGTTCCCGA AGGTTCTCCTCCCCGCTCT CATGATCTGCCCATCTTCTTGT	123 109 149 147 100 134

### Western Blot Analysis

Cells were lysed in RIPA buffer (Solarbio, Beijing, China) for 30 min on ice, and then centrifuged at 12,000 *g* for 10 min at 4°C. The concentrations of proteins were measured with a BCA kit (Takara, Dalian, China). About 30 μg total proteins from each sample were separated by polyacrylamide gel electrophoresis in the presence of sodium dodecyl sulfate (SDS-PAGE) and transferred onto the poly-vinylidene fluoride (PVDF) membrane at 10 V using the Bio-Rad semidry transfer system. The blot-transferred membranes were blocked with 5% fat-free dry milk dissolved in Tris-buffered saline containing 0.1% tween-20 (TBST) for 2 h and then incubated with primary antibodies (Table 1) on the shaker at 4°C overnight. Blots were washed with TBST, followed by incubation with a horseradish peroxidase (HRP)-conjugated anti-rabbit or anti-mouse IgG antibody (Millipore). Protein bands were visualized under a Bio-Rad Chemidoc XRS with a Western Bright ECL Kit (Bio-Rad, Berkeley, CA, United States) and digital images were captured. Finally, the gray scale analysis was performed by comparing the signals of target proteins with those of the housekeeper β-actin.

### Statistics

Experimental data were analyzed with the Graph Pad Prism 6 software and presented as the mean ± SEM. A Student’s *t*-test (two-sided) or one-way ANOVA followed by a Duncan’s multiple range test (SPSS 19.0; Chicago, IL, United States) was performed to determine the significant value. A value of *p* < 0.05 was considered statistically significant. *p < 0.05; **p < 0.01.

## Results

### Cr (VI) Damaged Male Reproductive System and SSCs/Progenitors in Mouse Testes

**Figure 1:**
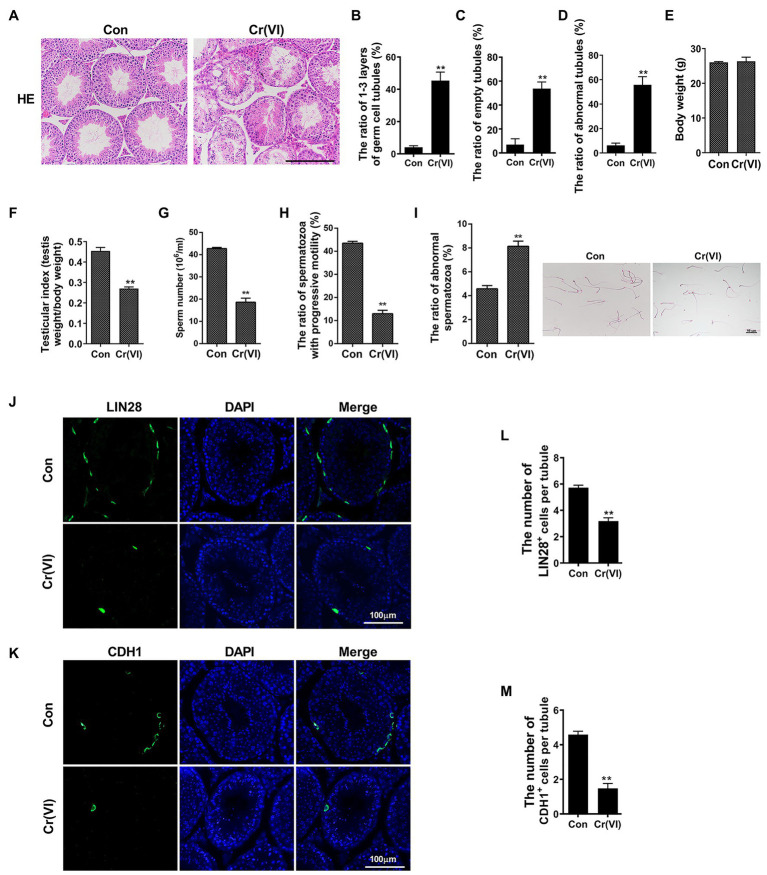
Chromium (Cr) (VI) damaged male reproductive system and spermatogonial stem cells (SSCs)/progenitors in mouse testes. **(A)** Hematoxylin and eosin (H&E) staining of testis sections from control and Cr (VI)-treated mice. Bar = 200 μm. **(B–D)** The ratios of only 1–3 layers of germ cell **(B)**, empty **(C)**, and abnormal **(D)** seminiferous tubules in control and Cr (VI)-treated mouse testes. **(E)** The average body weight in control and Cr (VI)-treated mice. **(F)** The average testicular index (testis weight/body weight) in control and Cr (VI)-treated mice. **(G)** The average sperm number (10^6^/ml) in control and Cr (VI)-treated mice. **(H)** The ratio of spermatozoa with progressive motility in control and Cr (VI)-treated mice. **(I)** Left: the ratio of abnormal spermatozoa in control and Cr (VI)-treated mice; Right: images of spermatozoa from the control and Cr (VI)-treated mice. Bar = 50 μm. **(J,K)** Immunofluorescence staining for LIN28 (J) and CDH1 (K) in testis sections from control and Cr (VI)-treated mice. Bar = 100 μm. **(L,M)** The numbers of LIN28^+^ **(L)** and CDH1^+^ cells **(M)** per seminiferous tubule in control and Cr (VI)-treated mouse testes. Data are presented as the mean ± SEM from five mice, and 300 seminiferous tubules from five mice were analyzed in each group. **p < 0.01.

To systematically investigate the toxic effects of Cr (VI) on male reproductive system, we randomly divided 20 male C57/BL6 mice into two groups: control and Cr (VI), with 10 mice in each group. Mice in the Cr (VI) treatment group were intraperitoneally injected with Cr (VI) (16.2 mg/kg *b.w.*/day) for 14 consecutive days. The dosage was based on previous reports (Kart et al., 2016; Ogbomida et al., 2018) and our preliminary experiment (data not shown). All mice in both groups were sacrificed on the 14th day after the last administration, and the testes were removed for examination. H&E staining results showed that seminiferous tubules were clearly diminished after Cr (VI) administration. Cr (VI) administration also triggered nuclear pyknosis and exfoliation of spermatogenic cells, as well as the emergence of different sizes of vacuoles and even cavities in some seminiferous tubules. Quantification analyses revealed markedly higher ratios of only 1–3 layers of germ cell, empty and abnormal tubules in the Cr (VI) treatment group (Figures 1A–D). In addition, despite no significant difference in body weight (Figure 1E), Cr (VI) treatment clearly decreased the testicular index (the ratio of testis weight to body weight, Figure 1F), the sperm number (Figure 1G), and progressive motility (Figure 1H), along with an increase of abnormal epididymal spermatozoa (Figure 1I).

Next, we performed immunofluorescence staining on testis sections with SSC/progenitor markers LIN28 (Zheng et al., 2009; Figure 1J) and CDH1 (Tokuda et al., 2007; Figure 1K). After quantification, we identified that the numbers of LIN28^+^ and CDH1^+^ cells per seminiferous tubule were significantly reduced after Cr (VI) treatment (Figures 1L,M), suggesting that Cr (VI) also induces damage to SSCs/progenitors.

### Cr (VI) Impaired Cell Viability and Induced ROS Generation in SSCs/Progenitors

**Figure 2:**
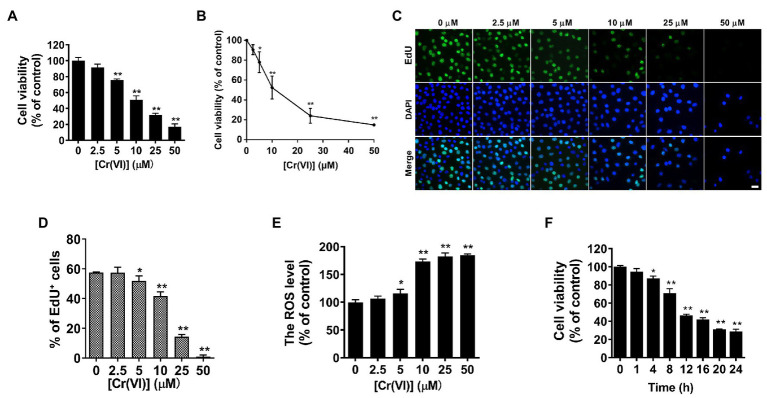
Chromium (VI) impaired cell viability and induced reactive oxygen species (ROS) generation in SSCs/progenitors. **(A,B)** A Cell Counting Kit-8 (CCK-8; **A**) and MTT assay **(B)** for the viability of SSCs/progenitors treated with different concentrations of Cr (VI) for 12 h. **(C)** Representative images of cells in different treatment groups that incorporate EdU. Bar = 20 μm. **(D)** Quantification of cells in different treatment groups that incorporate EdU. At least 300 cells were analyzed in each group. **(E)** DCFH-DA detection of the intracellular ROS accumulation in SSCs/progenitors treated with different concentrations of Cr (VI) for 1 h. **(F)** A CCK-8 assay for the viability of SSCs/progenitors treated with 10 μM Cr (VI) for different time. Data are presented as the mean ± SEM of three independent experiments. *p < 0.05; **p < 0.01.

Previous articles described that the cell survival rate was significantly decreased when exposed to 12.5 μM or higher concentrations of Cr (VI) (Hu et al., 2016, 2018). To investigate the influence of Cr (VI) on SSC viability, we employed an immortalized mouse SSC/progenitor line, i.e., C-184 (Hofmann et al., 2005). These cells were exposed to different dosages of Cr (VI), and then subjected to a CCK-8 assay. As shown in Figure 2A, treatment of Cr (VI) for 12 h decreased the percentages of viable cells in a concentration-dependent manner, with an approximately 50% of decrease in the 10 μM Cr (VI) treatment group. MTT (Figure 2B) and EdU assays (Figures 2C,D) generated similar results. We also detected ROS generation under different Cr (VI) dosages. Consistently, the ROS level was significantly increased after 1 h of 10 μM Cr (VI) treatment (Figure 2E). We further incubated the cells with 10 μM Cr (VI) for different time. As expected, the cell viability was decreased in a time-dependent manner (Figure 2F). Thus, we applied 10 μM Cr (VI) treatment in subsequent *in vitro* experiments, unless otherwise stated. The overall data demonstrate that Cr (VI) impairs cell viability and induces ROS generation in SSCs/progenitors.

### Cr (VI) Induced Mitochondrial Dynamic Imbalance and Mitophagy in SSCs/Progenitors

**Figure 3:**
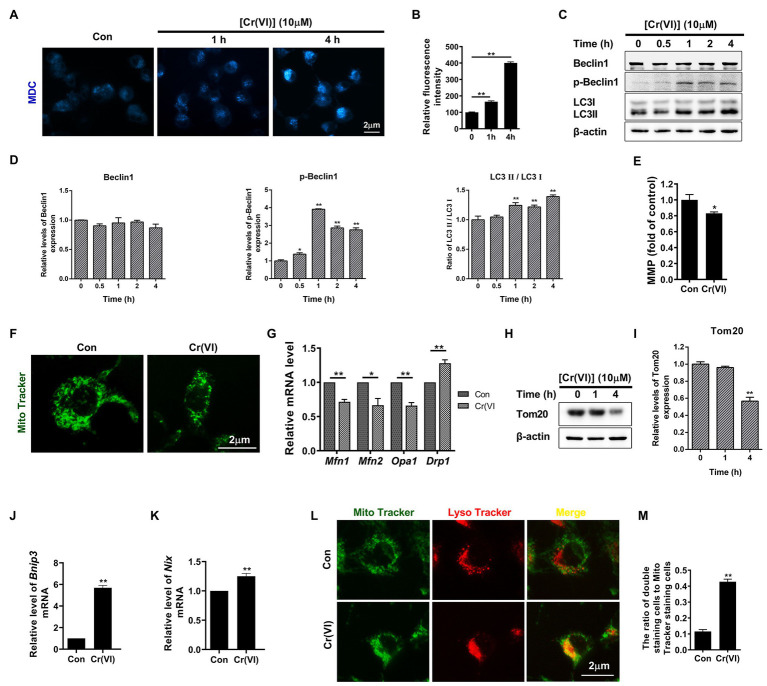
Chromium (VI) induced mitochondrial dynamic imbalance and mitophagy in SSCs/progenitors. **(A)** Monodansylcadaverine (MDC) staining for autophagic vacuoles. Bar = 2 μm. **(B)** The relative fluorescence intensity of MDC in SSCs/progenitors treated with 10 μM Cr (VI) for different time. **(C)** Western blot analysis of the expression levels of autophagy markers Beclin1, p-Beclin1, and LC3 in SSCs/progenitors treated with 10 μM Cr (VI) for different time. β-actin is used as a loading control. **(D)** The relative band intensities of Beclin1, p-Beclin1, and LC3-II in different cell treatment groups. **(E)** Mitochondrial membrane potential (MMP) in SSCs/progenitors treated with vehicle or 10 μM Cr (VI) for 4 h, as detected by the lipophilic cationic dye JC-1. **(F)** Mito Tracker staining for mitochondria in SSCs/progenitors treated with vehicle or 10 μM Cr (VI) for 4 h. Bar = 2 μm. **(G)** qPCR analysis of *Mfn1*, *Mfn2*, *Opa1*, and *Drp1* in SSCs/progenitors treated with vehicle or 10 μM Cr (VI) for 4 h. **(H)** Western blot analysis of Tom20 in SSCs/progenitors treated with 10 μM Cr (VI) for different time. β-actin is used as a loading control. **(I)** The relative band intensities of Tom20 in different cell treatment groups. **(J,K)** qPCR analysis of *Bnip3* **(J)** and *Nix* **(K)** in SSCs/progenitors treated with vehicle or 10 μM Cr (VI) for 4 h. **(L)** Mito and Lyso Tracker co-staining analysis in SSCs/progenitors treated with vehicle or 10 μM Cr (VI) for 4 h. Bar = 2 μm. **(M)** The ratios of double staining cells to Mito Tracker staining cells in the control and 4 h of 10 μM Cr (VI) treatment group. Data are presented as the mean ± SEM of three independent experiments. *p < 0.05; **p < 0.01.

Monodansylcadaverine, a fluorescent pigment, is typically used to detect the occurrence of autophagy (Weiss-Sadan et al., 2019; de Campos et al., 2020). We identified that the fluorescence intensity of MDC was clearly increased after 10 μM Cr (VI) treatment (Figures 3A,B), suggestive of Cr (VI)-induced autophagy in SSCs/progenitors. We then investigated whether autophagy-associated genes were also activated by Cr (VI). To this end, we incubated mouse SSCs/progenitors with 10 μM Cr (VI) for different time. As shown in Figures 3C,D, p-Beclin1 and LC3-II, two autophagy markers, were upregulated by Cr (VI) in a time-dependent manner.

In addition to ROS generation (Figure 2E), we found that treatment with 10 μM Cr (VI) for 4 h also decreased mitochondrial membrane potential (Figure 3E). Excessive ROS production and decreased MMP could indicate malfunctioned mitochondria. Hence, we performed Mito Tracker staining, and found that Cr (VI) caused mitochondrial aggregation and displaying the short rod morphology (Figure 3F). We assumed that Cr (VI) might disturb the balance between mitochondrial fusion and fission. To this end, we performed a qPCR analysis for mitochondrial fusion- (*Mfn1*, *Mfn2*, and *Opa1*) and fission-related genes (*Drp1*; Varuzhanyan and Chan, 2020). The qPCR result showed significant downregulation of *Mfn1*, *Mfn2*, and *Opa1* but upregulation of *Drp1* at the mRNA level (Figure 3G). Besides, the mitochondrial marker Tom20, a protein that localizes on the mitochondrial outer membrane (OMM) and responsible for the first step of mitochondrial protein transportation (Di Maio et al., 2016), was substantially downregulated after 4 h of 10 μM Cr (VI) treatment, as demonstrated by the Western blot (WB) analysis (Figures 3H,I).

**Figure 4:**
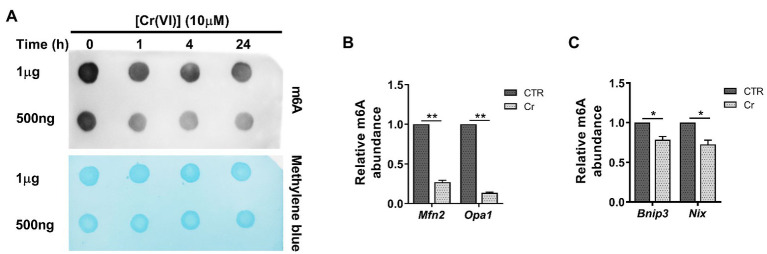
Chromium (VI) downregulated the RNA N^6^-methyladenosine (m^6^A) modification levels in mitochondrial dynamic balance and mitophagy genes in SSCs/progenitors. **(A)** The m^6^A dot-blot assay showing the global RNA m^6^A modification levels in SSCs/progenitors treated with 10 μM Cr (VI) for different time. About 1 μg means 1 μg mRNAs, and 500 ng means 500 ng extracted and purified mRNAs from SSCs/progenitors. Methylene blue is used as a loading control to eliminate the difference in mRNA amount. **(B,C)** The m^6^A-IP-qPCR analysis showing the relative m^6^A abundance in mitochondrial fusion genes *Mfn2*, *Opa1* **(B)** and in mitophagy genes *Bnip3*, *Nix* **(C)** in SSCs/progenitors treated with vehicle or 10 μM Cr (VI) for 4 h. Data are presented as the mean ± SEM of three independent experiments. *p < 0.05; **p < 0.01.

To investigate whether Cr (VI) induces mitophagy to clear damaged mitochondria in SSCs, we conducted a qPCR analysis for mitophagy activation genes *Bnip3* and *Nix* (Lampert et al., 2019). As shown in Figures 3J,K, *Bnip3* and *Nix* were significantly upregulated after 4 h of 10 μM Cr (VI) treatment. We further detected the ratio of autophagic lysosome (AL)-engulfed mitochondria, with Mito and Lyso Tracker staining to label mitochondria and lysosomes, respectively. As shown in Figures 3L,M, 4 h of 10 μM Cr (VI) treatment considerably increased the ratio of cells double positive for both Mito and Lyso staining, suggesting the elevated number of cells with impaired mitochondria that are engulfed by ALs.

### Cr (VI) Downregulated the RNA m^6^A Modification Levels in Mitochondrial Dynamic Balance and Mitophagy Genes in SSCs/Progenitors

Next, we investigated whether Cr (VI) affects the m^6^A modification in SSCs. To this end, we treated mouse SSCs/progenitors with 10 μM Cr (VI), followed by RNA extraction and m^6^A dot-blot to detect the m^6^A level. As shown in Figure 4A, the RNA m^6^A modification level showed a clear decrease after 1 h of Cr (VI) treatment, and then remained stable. To investigate whether RNA m^6^A modification is involved in Cr (VI)-induced mitochondrial dynamic imbalance and mitophagy in SSCs, we additionally performed a m^6^A-IP-qPCR assay for mitochondrial fusion and mitophagy genes. The result uncovered that 4 h of 10 μM Cr (VI) treatment reduced the m^6^A modification levels in mitochondrial fusion genes *Mfn2* and *Opa1* (Figure 4B), as well as in mitophagy genes *Bnip3* and *Nix* (Figure 4C), suggesting potential roles of RNA m^6^A modification in Cr (VI)-induced mitochondrial abnormality in SSCs/progenitors.

### Melatonin Alleviated Cr (VI)-Induced Damage to Male Reproductive System and Autophagy in Mouse Testes

**Figure 5:**
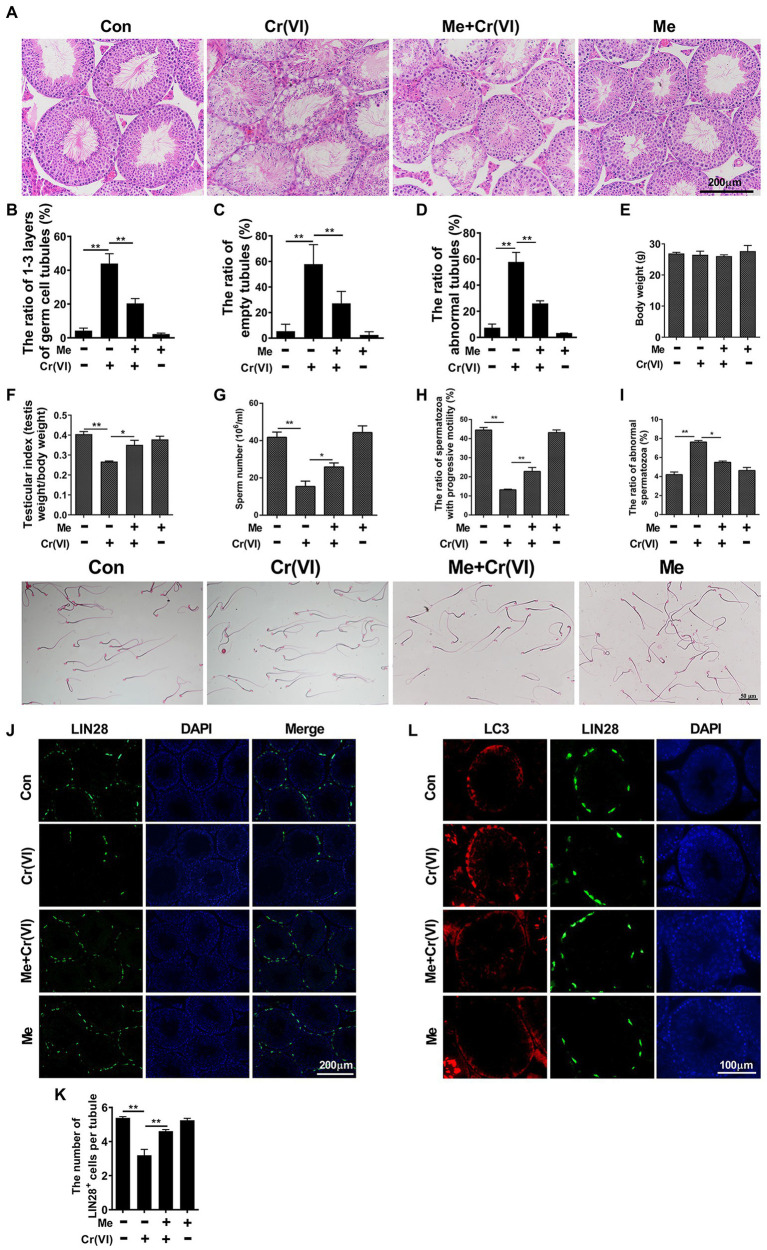
Melatonin alleviated Cr (VI)-induced damage to male reproductive system and autophagy in mouse testes. **(A)** H&E staining of testis sections from the control, Cr (VI), melatonin + Cr (VI), and melatonin-treated mice. Bar = 200 μm. **(B–D)** The ratios of only 1–3 layers of germ cell **(B)**, empty **(C)**, and abnormal **(D)** seminiferous tubules in testes from the control, Cr (VI), melatonin + Cr (VI), and melatonin-treated mice. **(E)** The average body weight in different mouse groups. **(F)** The average testicular index (testis weight/body weight) in different mouse groups. **(G)** The average sperm number (10^6^/ml) in different mouse groups. **(H)** The ratio of spermatozoa with progressive motility in different mouse groups. **(I)** The upper panel: the ratio of abnormal spermatozoa in different mouse groups; The lower panel: images of spermatozoa from different groups of mice. Bar = 50 μm. **(J)** Immunofluorescence staining for LIN28 in testis sections from the control, Cr (VI), melatonin + Cr (VI), and melatonin-treated mice. Bar = 200 μm. **(K)** The numbers of LIN28^+^ cells per seminiferous tubule in testes from the control, Cr (VI), melatonin + Cr (VI), and melatonin-treated mice. **(L)** Co-staining analysis for LC3 and LIN28 in testis sections from the control, Cr (VI), melatonin + Cr (VI), and melatonin-treated mice. Bar = 100 μm. Data are presented as the mean ± SEM from five mice, and 300 seminiferous tubules from five mice were analyzed in each group. *p < 0.05; **p < 0.01.

Melatonin is a strong endogenous free radical scavenger of ROS and inducer of antioxidant systems *in vivo* (Bonnefont-Rousselot et al., 2011). To explore whether melatonin has protective roles against Cr (VI)-induced testicular damage, we randomly divided 40 male C57/BL6 mice into four groups: control, Cr (VI), melatonin + Cr (VI), melatonin, with 10 mice in each group. In the melatonin + Cr (VI) group, the mice were pre-intraperitoneally injected with melatonin (25 mg/kg *b.w.*/day), followed by intraperitoneal injection with Cr (VI; 16.2 mg/kg *b.w.*/day). Melatonin and Cr (VI) treatment lasted for 14 consecutive days. The mice were sacrificed on the 14th day after the last treatment, and the testes were removed for examination. H&E staining results showed that melatonin treatment effectively attenuated Cr (VI)-induced damage to testes, characterized by alleviated vacuolization of seminiferous tubules, reduced ratios of only 1–3 layers of germ cell, empty and abnormal tubules (Figures 5A–D). In addition, despite no significant difference in body weight (Figure 5E), melatonin pretreatment restored the testicular index (Figure 5F), sperm number (Figure 5G), and progressive motility (Figure 5H), along with diminished abnormal epididymal spermatozoa (Figure 5I).

The subsequent immunofluorescence staining analysis uncovered that melatonin treatment also attenuated Cr (VI)-induced decrease of LIN28^+^ cells per seminiferous tubule (Figures 5J,K), suggesting the preservation of SSCs/progenitors by melatonin. Besides, the autophagy marker LC3-II was less induced with melatonin treatment (Figure 5L). The overall data therefore suggest that melatonin could alleviate Cr (VI)-induced damage to male reproductive system and autophagy in mouse testes.

### Melatonin Attenuated Cr (VI)-Induced Cell Viability Loss and ROS Generation in SSCs/Progenitors

**Figure 6:**
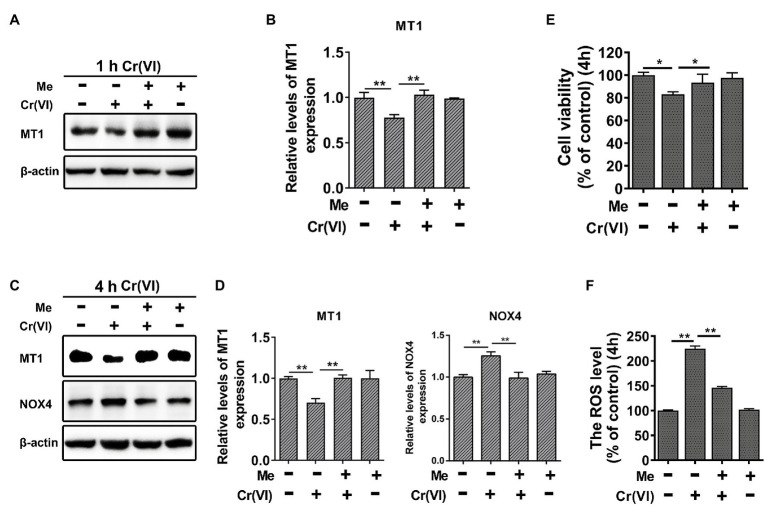
Melatonin attenuated Cr (VI)-induced cell viability loss and ROS generation in SSCs/progenitors. **(A)** Western blot analysis of melatonin receptor 1 (MT1) in the control, Cr (VI), melatonin + Cr (VI), and melatonin-treated SSCs/progenitors. Of these, Cr (VI) treatment lasted for 1 h. β-actin is used as a loading control. **(B)** The relative expression of MT1 in different cell groups after 1 h of Cr (VI) treatment. **(C)** Western blot analysis of MT1 and NADPH oxidase 4 (NOX4) in the control, Cr (VI), melatonin + Cr (VI), and melatonin-treated SSCs/progenitors. Of these, Cr (VI) treatment lasted for 4 h. β-actin is used as a loading control. **(D)** The relative expression of MT1 and NOX4 in different cell groups after 4 h of Cr (VI) treatment. **(E)** A CCK-8 assay for SSC/progenitor viability in the control, Cr (VI), melatonin + Cr (VI), and melatonin treatment group. Of these, Cr (VI) treatment lasted for 4 h. **(F)** DCFH-DA detection of the intracellular ROS accumulation in the control, Cr (VI), melatonin + Cr (VI), and melatonin-treated SSCs/progenitors. Of these, Cr (VI) treatment lasted for 4 h. Data are presented as the mean ± SEM of three independent experiments. *p < 0.05; **p < 0.01.

Then, we explored the protective roles of melatonin against Cr (VI)-induced oxidative damage in SSCs. Mouse SSCs/progenitors were pretreated with 50 μM melatonin for 2 h, followed by treatment with 10 μM Cr (VI) for 1 or 4 h. By WB analysis, we found that while 1 h of Cr (VI) treatment downregulated melatonin receptor 1 (MT1), pre-treatment with melatonin could alleviate this (Figures 6A,B). In addition, NADPH oxidase 4 (NOX4), a protein that is expressed in mitochondria and mediates ROS production (Kuroda et al., 2010), was upregulated after 4 h of Cr (VI) treatment, but pretreatment with melatonin relieved its upregulation (Figures 6C,D). Similarly, cell viability loss and ROS generation, both of which were induced by 4 h of Cr (VI) treatment, could be attenuated by pretreatment with melatonin (Figures 6E,F).

### Melatonin Attenuated Cr (VI)-Induced Mitochondrial Dynamic Disorders and Mitophagy *via* RNA m^6^A Modification in SSCs/Progenitors

**Figure 7:**
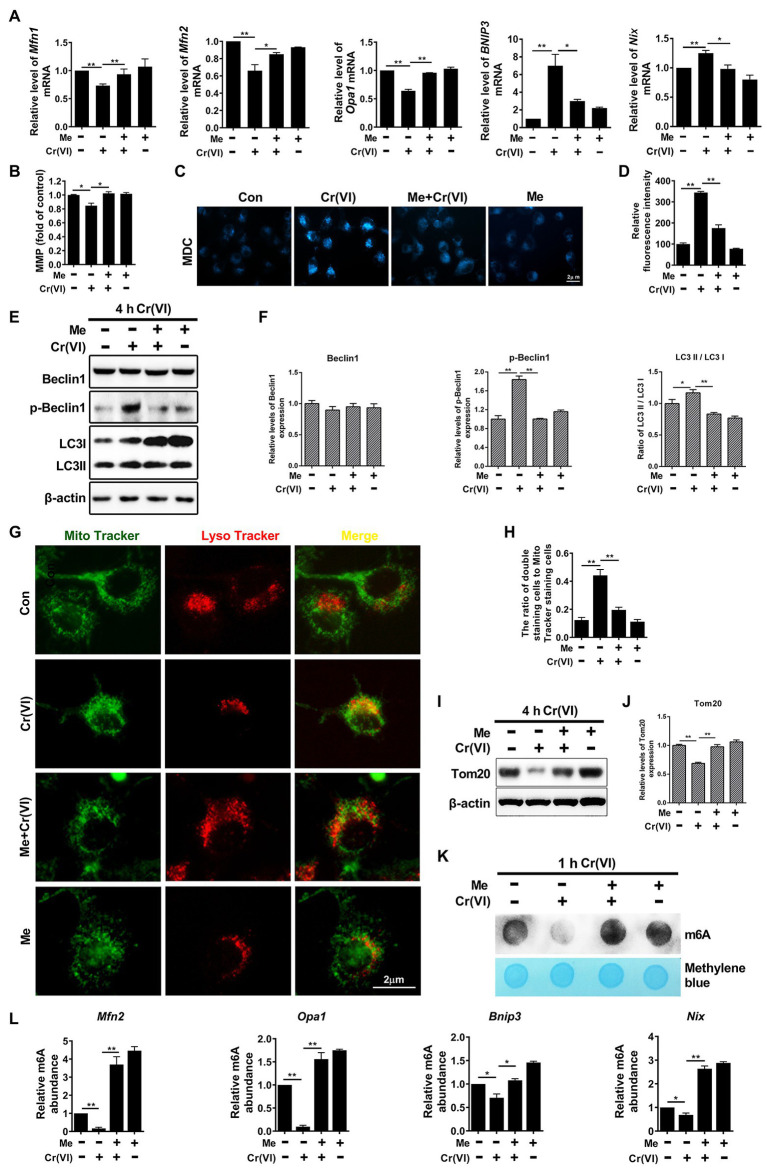
Melatonin attenuated Cr (VI)-induced mitochondrial dynamic disorders and mitophagy *via* RNA m^6^A modification in SSCs/progenitors. **(A)** qPCR analysis of *Mfn1*, *Mfn2*, *Opa1*, *Bnip3*, and *Nix* in the control, Cr (VI), melatonin + Cr (VI), and melatonin-treated SSCs/progenitors. **(B)** MMP in the control, Cr (VI), melatonin + Cr (VI), and melatonin-treated SSCs/progenitors, as detected by the lipophilic cationic dye JC-1. **(C)** MDC staining for autophagic vacuoles in the control, Cr (VI), melatonin + Cr (VI), and melatonin-treated SSCs/progenitors. Bar = 2 μm. **(D)** The relative fluorescence intensity of MDC in the control, Cr (VI), melatonin + Cr (VI), and melatonin-treated SSCs/progenitors. **(E)** Western blot analysis of the expression levels of autophagy markers Beclin1, p-Beclin1, and LC3 in the control, Cr (VI), melatonin + Cr (VI), and melatonin-treated SSCs/progenitors. Of these, Cr (VI) treatment lasted for 4 h. β-actin is used as a loading control. **(F)** The relative band intensities of Beclin1, p-Beclin1, and LC3-II in different cell treatment groups. **(G)** Mito and Lyso Tracker co-staining analysis in the control, Cr (VI), melatonin + Cr (VI), and melatonin-treated SSCs/progenitors. Bar = 2 μm. **(H)** The ratios of double staining cells to Mito Tracker staining cells in the control, Cr (VI), melatonin + Cr (VI), and melatonin-treated SSCs/progenitors. **(I)** Western blot analysis of Tom20 in the control, Cr (VI), melatonin + Cr (VI), and melatonin-treated SSCs/progenitors. Of these, Cr (VI) treatment lasted for 4 h. β-actin is used as a loading control. **(J)** The relative band intensities of Tom20 in different cell treatment groups. **(K)** The m^6^A dot-blot assay showing the global RNA m^6^A modification levels in the control, Cr (VI), melatonin + Cr (VI), and melatonin-treated SSCs/progenitors. Of these, Cr (VI) treatment lasted for 1 h. Methylene blue is used as a loading control to eliminate the difference in mRNA amount. **(L)** The m^6^A-IP-qPCR analysis showing the relative m^6^A abundance in mitochondrial fusion genes *Mfn2*, *Opa1* and in mitophagy genes *Bnip3*, *Nix* in the control, Cr (VI), melatonin + Cr (VI), and melatonin-treated SSCs/progenitors. Data are presented as the mean ± SEM of three independent experiments. *p < 0.05; **p < 0.01.

Next, we probed the influence of melatonin on Cr (VI)-induced mitochondrial abnormality in SSCs. Mouse SSCs/progenitors were still pretreated with 50 μM melatonin for 2 h, followed by treatment with 10 μM Cr (VI) for 4 h. The qPCR result showed that pretreatment with melatonin maintained the mRNA levels of mitochondrial fusion genes *Mfn1*, *Mfn2*, and *Opa1*, while counteracted Cr (VI)-induced upregulation of mitophagy genes *Bnip3* and *Nix* (Figure 7A). MMP was also restored by melatonin pretreatment (Figure 7B). Moreover, pretreatment with melatonin attenuated Cr (VI)-induced increase of the MDC fluorescence intensity (Figures 7C,D) and upregulation of autophagy markers p-Beclin1 and LC3-II (Figures 7E,F), as revealed by MDC staining for autophagic vacuoles and Western blot analysis, respectively. In addition, melatonin pretreatment attenuated Cr (VI)-induced increase of cells double positive for both Mito and Lyso staining (Figures 7G,H), i.e., cells with impaired mitochondria that are engulfed by ALs. Melatonin pretreatment was also found to maintain the protein level of the mitochondrial marker Tom20 (Figures 7I,J).

Later, we performed a m^6^A dot-blot analysis to investigate whether melatonin affects RNA m^6^A modification in SSCs. The m^6^A dot-blot result showed that melatonin pretreatment restored the RNA m^6^A level that was decreased after 1 h of Cr (VI) treatment (Figure 7K). We further conducted a m^6^A-IP-qPCR assay for mitochondrial fusion and mitophagy genes. Interestingly, it was identified that melatonin pretreatment reversed the Cr (VI)-induced decrease of m^6^A modification levels in mitochondrial fusion genes *Mfn2* and *Opa1*, as well as in mitophagy genes *Bnip3* and *Nix* (Figure 7L). Therefore, the overall data suggest that melatonin could attenuate Cr (VI)-induced mitochondrial dynamic disorders and mitophagy *via* RNA m^6^A modification in SSCs/progenitors.

### Melatonin Attenuated Cr (VI)-Induced Decrease of the RNA m^6^A Modification Level *via* METTL3 in SSCs/Progenitors

**Figure 8:**
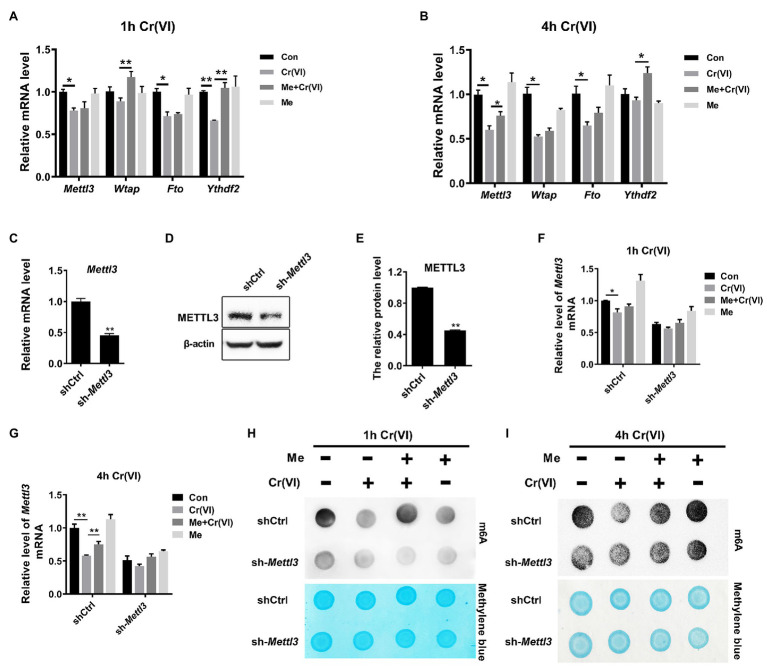
Melatonin attenuated Cr (VI)-induced decrease of the RNA m^6^A modification level *via* METTL3 in SSCs/progenitors. **(A,B)** qPCR analysis of *Mettl3*, *Wtap*, *Fto*, and *Ythdf2* in the control, Cr (VI), melatonin + Cr (VI), and melatonin-treated SSCs/progenitors. Of these, Cr (VI) treatment lasted for 1 h **(A)** or 4 h **(B)**. **(C,D)** qPCR **(C)** and Western blot analysis **(D)** of METTL3 in the scramble control and *Mettl3*-shRNA SSC/progenitor group. β-actin is used as a loading control. **(E)** The METTL3 band intensities relative to β-actin in the scramble control and *Mettl3*-shRNA SSC/progenitor group. **(F,G)** qPCR analysis of *Mettl3* in the scramble control and *Mettl3*-shRNA SSCs/progenitors treated with vehicle, Cr (VI), melatonin + Cr (VI), or melatonin. Of these, Cr (VI) treatment lasted for 1 h **(F)** or 4 h **(G)**. **(H,I)** The m^6^A dot-blot assay showing the global RNA m^6^A modification levels in the scramble control and *Mettl3*-shRNA SSCs/progenitors treated with vehicle, Cr (VI), melatonin + Cr (VI), or melatonin. Of these, Cr (VI) treatment lasted for 1 **(H)** or 4 h **(I)**. Methylene blue is used as a loading control to eliminate the difference in mRNA amount. Data are presented as the mean ± SEM of three independent experiments. *p < 0.05; **p < 0.01.

We subsequently delved into the mechanisms underlying melatonin-restored RNA m^6^A modification levels that were reduced by Cr (VI). To this end, we carried out a qPCR analysis for m^6^A-associated genes, i.e., *Mettl3*, *Wtap*, *Fto*, and *Ythdf2*, encoding methyltransferases METTL3, WTAP (the m^6^A writers), the demethylase FTO (the m^6^A eraser) and the m^6^A-binding protein YTHDF2 (the m^6^A reader), respectively (Shi et al., 2019). By qPCR analysis, we found that *Mettl3* was decreased at the mRNA level after 4 h of Cr (VI) treatment, but that pretreatment with melatonin attenuated the Cr (VI)-induced downregulation of *Mettl3* (Figures 8A,B).

METTL3 is a major component of the m^6^A methyltransferase complex (Shi et al., 2019). As Cr (VI) downregulated the global RNA m^6^A modification level and *Mettl3*, but both could be maintained by melatonin pretreatment in SSCs/progenitors, we then explored whether melatonin attenuates the Cr (VI)-decreased RNA m^6^A modification level *via* METTL3. To this end, we constructed a *Mettl3*-depleted mouse SSC/progenitor line by lentivirus-mediated shRNA targeting. qPCR and Western blot results demonstrated significant downregulation of METTL3 (Figures 8C–E). Subsequently, the *Mettl3*-depleted and control cell lines were pretreated with 50 μM melatonin, followed by treatment with 10 μM Cr (VI) for 1 or 4 h. The qPCR analysis revealed that while the Cr (VI)-induced downregulation of *Mettl3* was attenuated by melatonin pretreatment in control cells, the *Mettl3* expression level was neither significantly reduced by Cr (VI) treatment nor restored by melatonin pretreatment in *Mettl3*-depleted cells (Figures 8F,G). The m^6^A dot-blot result demonstrated the overall reduced m^6^A modification level by *Mettl3* depletion and, notably, that while the Cr (VI)-induced decrease of the RNA m^6^A modification level was attenuated by melatonin pretreatment in control cells, this could not be recapitulated in *Mettl3*-depleted cells (Figures 8H,I). Hence, the overall data suggest that melatonin could attenuate Cr (VI)-induced decrease of the RNA m^6^A modification level *via* METTL3 in SSCs/progenitors.

### Melatonin Attenuated Cr (VI)-Induced Mitophagy by Restoration of METTL3-Mediated RNA m^6^A Modification in SSCs/Progenitors

**Figure 9:**
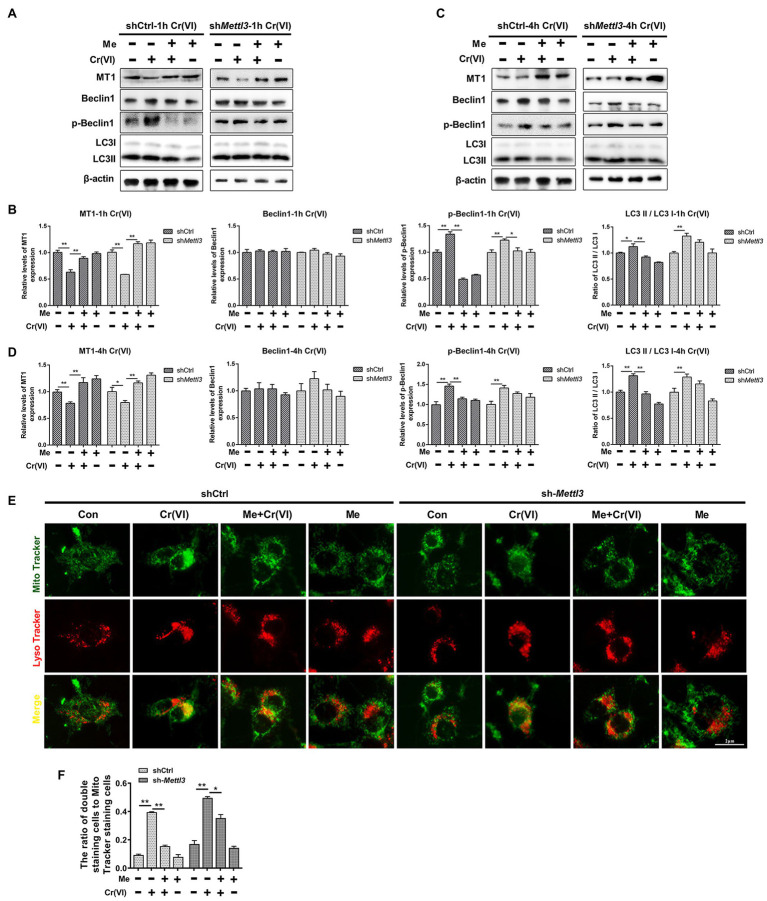
Melatonin attenuated Cr (VI)-induced mitophagy by restoration of METTL3-mediated RNA m^6^A modification in SSCs/progenitors. **(A,C)** Western blot analysis of MT1, Beclin1, p-Beclin1, and LC3 in the scramble control and *Mettl3*-shRNA SSCs/progenitors treated with vehicle, Cr (VI), melatonin + Cr (VI), or melatonin. Of these, Cr (VI) treatment lasted for 1 **(A)** or 4 h **(C)**. β-actin is used as a loading control. **(B,D)** The relative expression of MT1, Beclin1, p-Beclin1, and LC3-II in different cell groups after 1 **(B)** or 4 h **(D)** of Cr (VI) treatment. **(E)** Mito and Lyso Tracker co-staining analysis in the scramble control and *Mettl3*-shRNA SSCs/progenitors treated with vehicle, Cr (VI), melatonin + Cr (VI), or melatonin. Of these, Cr (VI) treatment lasted for 4 h. Bar = 2 μm. **(F)** The ratios of double staining cells to Mito Tracker staining cells in the scramble control and *Mettl3*-shRNA SSCs/progenitors treated with vehicle, Cr (VI), melatonin + Cr (VI), or melatonin. Data are presented as the mean ± SEM of three independent experiments. *p < 0.05; **p < 0.01.

Finally, we investigated whether melatonin could attenuate Cr (VI)-induced mitophagy in SSCs in the same way, i.e., by restoration of METTL3-mediated RNA m^6^A modification. Still, the *Mettl3*-depleted and control cell lines were pretreated with 50 μM melatonin, followed by treatment with 10 μM Cr (VI) for 1 h or 4 h. Western bolt analysis revealed that while the expression level of MT1 was maintained by melatonin pretreatment in both cell lines, Cr (VI)-induced autophagy markers p-Beclin1 and LC3-II were only minimally downregulated in melatonin-pretreated *Mettl3*-depleted cells (Figures 9A–D). Similarly, melatonin pretreatment relatively less attenuated Cr (VI)-induced increase of cells double positive for both Mito and Lyso staining (cells with impaired mitochondria that are engulfed by ALs) in *Mettl3*-depleted cells (Figures 9E,F). Together, these data suggest that melatonin could attenuate Cr (VI)-induced mitophagy by restoration of METTL3-mediated RNA m^6^A modification in SSCs/progenitors.

## Discussion

Chromium (VI) is a proven toxin, mutagen, and carcinogen. Although the adverse effects of Cr (VI) on fitness have appealed to the public, its deleterious effects on male fertility in particular SSCs remain poorly understood. In this study, we identified that exposure of SSCs/progenitors to 10 μM Cr (VI) for 4 h upregulated LC3-II and p-Beclin1. LC3-II and p-Beclin1 are two well-known markers for autophagy. Autophagy is a universal event occurring in eukaryotic cells to maintain homeostasis and metabolic balance (Mizushima et al., 2008). Technically, it is a conservative autophagic lysosomal degradation process characterized by transporting toxic proteins and damaged organelles into lysosomes to form autophagosomes, indispensable for cell elimination, reconstruction, growth, and development (Klionsky and Emr, 2000). During this process, the cytosolic LC3 (LC3-I) is converted to the autophagosomal membrane type (LC3-II). The presence of LC3 in autophagosomes and the conversion of LC3-I to LC3-II have thus been used as indicators of autophagy (Kabeya et al., 2000, 2004). In addition, phosphorylation of Beclin1 (p-Beclin1), a mammalian autophagy effector involved in autophagic vesicle nucleation, is important to sufficient autophagy induction (Aita et al., 1999; Liang et al., 1999). Therefore, upregulation of LC3-II and p-Beclin1 after Cr (VI) treatment indicates Cr (VI)-induced autophagy in SSCs/progenitors.

Mitochondria are highly dynamic organelles. In response to external stimuli, mitochondria frequently undergo fusion and fission (mitochondrial dynamics) to maintain dynamic balance thereby safeguarding normal cellular functions in various biological processes (Varuzhanyan and Chan, 2020). Mitochondrial fusion occurs *via* two distinct steps, both mediated by the large GTP-hydrolyzing enzymes of the dynamin superfamily. Of these, MFN1 and MFN2 mediate fusion of the mitochondrial OMM, while OPA1 mediates fusion of the inner membrane (IMM). Mitochondrial fusion is counterbalanced by fission that is regulated by DRP1 (Varuzhanyan and Chan, 2020). The appropriate dynamic balance between mitochondrial fusion and fission maintains the mitochondrial size, quantity, shape, and length. A recent article reported that clusterin relieved Cr (VI)-induced mitochondrial apoptosis in L02 hepatocytes by repressing the Ca^2+^-ROS-Drp1-mitochondrial fission axis (Tang et al., 2020). Despite that, little is known about the Cr (VI)-induced changes in mitochondrial dynamics in SSCs, if any. Here, we found that following Cr (VI) treatment, mitochondrial fusion genes *Mfn1*, *Mfn2*, and *Opa1* were significantly downregulated, while the key mitochondrial fission gene *Drp1* was upregulated. Besides, the short rod-like morphology indicative of mitochondrial aggregation was observed after Cr (VI) treatment. These, along with downregulation of the mitochondrial marker Tom20, excessive ROS production and decreased MMP, well demonstrate that Cr (VI) perturbs mitochondrial dynamics and homeostasis.

Typically, damaged mitochondria require elimination to maintain the cellular homeostasis, which is attained by mitophagy. Mitophagy, namely mitochondrial autophagy, is a targeted defense against mitochondrial impairment, playing critical roles in selective degradation of damaged or redundant mitochondria (Zhang et al., 2020b). There are two well-known routes for mitophagy: (1) the phosphatase and tensin homolog (PTEN) induced kinase I (PINK1)-Parkin pathway; and (2) the receptor (NIX/BNIP3L, BNIP3 and FUNDC1)-mediated pathway (Kubli and Gustafsson, 2012). Here, we found that exposure of SSCs/progenitors to 10 uM Cr (VI) for 4 h did not activate the PINK1-Parkin pathway. Instead, Cr (VI) treatment upregulated *Bnip* and *Nix*, suggesting induction of the receptor-mediated mitophagy pathway.

N^6^-methyladenosine is the most abundant internal modification of mRNAs in eukaryotes. In mammals, each mRNA contains an average of 3–5 m^6^A modifications within a consensus sequence (Meyer et al., 2012). The methylation and demethylation of m^6^A are dynamically regulated by m^6^A writers (methyltransferases, e.g., METTL3/14 and WTAP, responsible for catalyzing the m^6^A modification of adenosine acid on mRNAs), erasers (demethylases, e.g., FTO and ALKBH5, responsible for demethylation of m^6^A modified bases), and readers (m^6^A-binding proteins, e.g., YTHDF2, responsible for recognition of the base of m^6^A modification and for activation of downstream regulatory pathways involved in RNA degradation and miRNA processing; Shi et al., 2019). RNA m^6^A modification plays important regulatory roles in various biological processes, such as the response to inflammatory, DNA damage, cell proliferation, and survival (Shi et al., 2019). Despite this, the influence of RNA m^6^A modification on the response to Cr (VI)-induced toxicity in SSCs has so far not been studied. Here, we identified that Cr (VI) treatment induced autophagy in SSCs/progenitors, along with a decrease in the global RNA m^6^A level, suggesting that RNA m^6^A modification may play a regulatory role in Cr (VI)-induced SSC/progenitor autophagy. Indeed, the m^6^A-IP-qPCR assay uncovered that Cr (VI) treatment reduced the m^6^A modification levels in mitochondrial fusion genes *Mfn2* and *Opa1*, as well as in mitophagy genes *Bnip3* and *Nix*, lending support to potential roles of RNA m^6^A modification in Cr (VI)-induced mitochondrial abnormality in SSCs/progenitors.

Melatonin is the strongest endogenous free radical scavenger, implicated in antioxidant systems and prevention of oxidative damage in cells. Melatonin can directly act on testes and alleviate testicular damage caused by oxidative stress, apoptosis, hyperthermia, and inflammation (Dong et al., 2020; Li et al., 2020b; Zhang et al., 2020a). In addition, melatonin has been reported to protect mitochondria by scavenging ROS, inhibiting the mitochondrial permeability transition pore (MPTP), activating uncoupling proteins (UCPs), and maintaining the optimal MMP, together maintaining mitochondrial homeostasis and preserving mitochondrial functions (Tan et al., 2016). Since one of the main reasons for Cr (VI)-induced cytotoxicity is elevated ROS that trigger oxidative stress, we assumed that melatonin might have protective roles against Cr (VI)-induced cytotoxicity in SSCs. As expected, melatonin pretreatment downregulated the Cr (VI)-induced increase of NOX4 *via* MT1, thereby alleviating ROS overproduction and preserving cell viability. Also, our results demonstrated that melatonin pretreatment attenuated Cr (VI)-induced mitochondrial dynamic disorders and mitophagy, providing novel insights into the mechanisms underlying the protective roles of melatonin against Cr (VI)-induced cytotoxicity in SSCs/progenitors. Yet, a point that should not be overlooked is that the SSC niche and testosterone secretion may change after Cr (VI) and/or melatonin treatment. This, and the roles it plays, if any, are stimulating topics in future research.

Intriguingly, our results suggest that the protective roles of melatonin against Cr (VI)-induced mitochondrial dynamic disorders and mitophagy in SSCs/progenitors also involve RNA m^6^A modification. To gain more knowledge in this respect, we analyzed the expression levels of four well-known m^6^A-associated genes, i.e., *Mettl3*, *Wtap*, *Fto*, and *Ythdf2*, which encode the m^6^A writer, eraser, or reader, and found that melatonin pretreatment attenuated the Cr (VI)-induced downregulation of *Mettl3*. METTL3 is a key component of the m^6^A methyltransferase complex (Shi et al., 2019). We thus presumed that melatonin could exert its protective roles *via* METTL3-mediated RNA m^6^A modification. To this end, we constructed a *Mettl3*-depleted mouse SSC/progenitor line. As expected, *Mettl3* depletion reduced the overall RNA m^6^A level, and weakened the protective roles of melatonin against the Cr (VI)-induced decrease of the RNA m^6^A level and mitophagy. Thus, our study has for the first time demonstrated that melatonin could attenuate Cr (VI)-induced mitophagy by restoration of METTL3-mediated RNA m^6^A modification in SSCs/progenitors.

In this study, we reported the involvement of METTL3-mediated RNA m^6^A modification in the protective roles of melatonin against Cr (VI)-induced mitophagy in SSCs/progenitors. Indeed, apart from METTL3, other methyltransferases, demethylases, or RNA-binding proteins may also function in this process, which warrants systematic investigation in future. Besides, we identified that both the m^6^A abundance and mRNA levels of mitochondrial fusion genes *Mfn2* and *Opa1* were reduced by Cr (VI) treatment. Differently, Cr (VI) decreased the m^6^A abundance but increased the mRNA levels of receptor-mediated mitophagy pathway genes *Bnip3* and *Nix*. Given that m^6^A is a ubiquitous RNA modification, orchestrating mRNA splicing, translation, stability, and degradation (Niu et al., 2013), it would be stimulating to delve into the m^6^A-mediated transcriptional regulation in these genes. Future studies in these regards would extend the knowledge about the roles of RNA m^6^A modification in mitochondrial physiology, providing clues for treatment of disorders resulted from mitochondrial malfunction.

**Figure 10:**
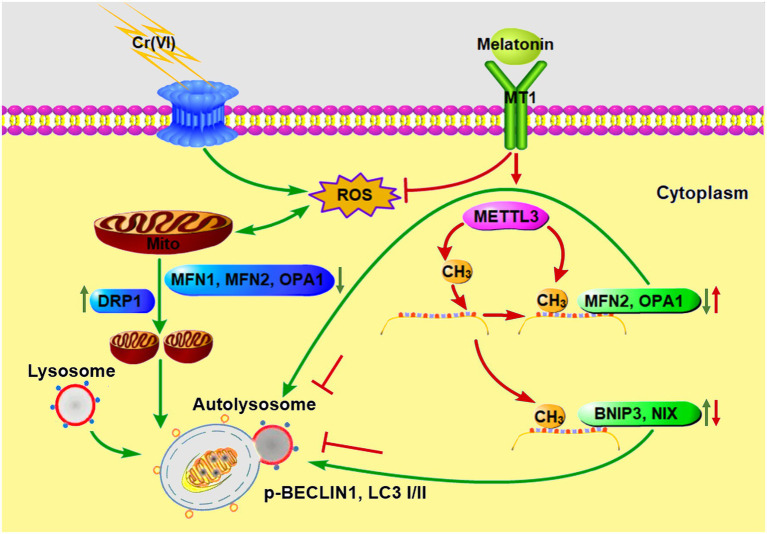
A schematic overview illustrating melatonin-mediated attenuation of Cr (VI)-induced mitochondrial dynamic disorders and mitophagy in SSCs. Cr (VI) exposure induces ROS generation and oxidative stress, and downregulates mitochondrial fusion proteins MFN1, MFN2, and OPA1, while upregulates the mitochondrial fission protein DRP1, resulting in mitochondrial dynamic disorders. The fragmented mitochondria fuse with lysosomes to form autolysosomes, causing aberrant mitophagy. Melatonin can inhibit ROS production and restore METTL3-mediated RNA m^6^A modification levels in mitochondrial fusion genes *Mfn2* and *Opa1*, as well as in mitophagy genes *Bnip3* and *Nix*, resulting in upregulation of MFN2 and OPA1 and downregulation of BNIP3 and NIX. In this way, autolysosomal formation and mitophagy are repressed. Green and red arrows point to Cr (VI)- and melatonin-induced biological processes, respectively.

To sum up, we identified that melatonin could attenuate Cr (VI)-induced mitochondrial dynamic disorders and mitophagy in SSCs/progenitors, *via* a mechanism illustrated in Figure 10. Our study does provide novel insights into the molecular mechanisms for RNA m^6^A modification underlying the gene regulatory network responsible for mitochondrial dynamic balance. Moreover, since SSCs are the cornerstone of spermatogenesis and able to differentiate into sperm thereby transmitting paternal genetic information to the next generation, our study, providing knowledge about the response of SSCs to environmental toxicant Cr (VI) and the underlying mechanisms for the protective roles of melatonin, would contribute to development of tailored therapies for Cr (VI)-induced damage to male fertility.

## Data Availability Statement

The original contributions presented in the study are included in the article/supplementary material; further inquiries can be directed to the corresponding authors.

## Ethics Statement

The animal study was reviewed and approved by Institutional Animal Care and Use Committee of Northwest A&F University.

## Author Contributions

YL and YZ conceived the study and designed the experiments. YL, TL, MY, LS, ZZ, and SZ performed the experiments. YL, TL, and YZ analyzed the data and wrote the manuscript. WZ and YZ supervised the study and approved the final submission. All authors contributed to the article and approved the submitted version.

### Conflict of Interest

The authors declare that the research was conducted in the absence of any commercial or financial relationships that could be construed as a potential conflict of interest.
